# Cetaceans are the next frontier for vocal rhythm research

**DOI:** 10.1073/pnas.2313093121

**Published:** 2024-05-30

**Authors:** Taylor A. Hersh, Andrea Ravignani, Hal Whitehead

**Affiliations:** ^a^Marine Mammal Institute, Oregon State University, Newport, OR 97365; ^b^Comparative Bioacoustics Group, Max Planck Institute for Psycholinguistics, Nijmegen 6525 XD, The Netherlands; ^c^Department of Biology, Dalhousie University, Halifax NS B3H 4R2, Canada; ^d^Center for Music in the Brain, Department of Clinical Medicine, Aarhus University, Aarhus 8000, Denmark; ^e^Department of Human Neurosciences, Sapienza University of Rome, Rome 00185, Italy

**Keywords:** bioacoustics, evolution of communication, isochrony, periodicity, timing

## Abstract

While rhythm can facilitate and enhance many aspects of behavior, its evolutionary trajectory in vocal communication systems remains enigmatic. We can trace evolutionary processes by investigating rhythmic abilities in different species, but research to date has largely focused on songbirds and primates. We present evidence that cetaceans—whales, dolphins, and porpoises—are a missing piece of the puzzle for understanding why rhythm evolved in vocal communication systems. Cetaceans not only produce rhythmic vocalizations but also exhibit behaviors known or thought to play a role in the evolution of different features of rhythm. These behaviors include vocal learning abilities, advanced breathing control, sexually selected vocal displays, prolonged mother–infant bonds, and behavioral synchronization. The untapped comparative potential of cetaceans is further enhanced by high interspecific diversity, which generates natural ranges of vocal and social complexity for investigating various evolutionary hypotheses. We show that rhythm (particularly isochronous rhythm, when sounds are equally spaced in time) is prevalent in cetacean vocalizations but is used in different contexts by baleen and toothed whales. We also highlight key questions and research areas that will enhance understanding of vocal rhythms across taxa. By coupling an infraorder-level taxonomic assessment of vocal rhythm production with comparisons to other species, we illustrate how broadly comparative research can contribute to a more nuanced understanding of the prevalence, evolution, and possible functions of rhythm in animal communication.

We live in a rhythmic world. From seasons and tides to heartbeats and brainwaves, rhythms orchestrate life. Rhythm is also an intrinsic component of many animal communication systems, including human (*Homo sapiens*) language and music, but the evolution and function of such patterns is unclear for most species. Here, we argue that cetaceans—whales, dolphins, and porpoises—are excellent but underutilized models for vocal rhythm research. Many cetaceans produce rhythmic vocalizations and exhibit behaviors that have been linked to vocal rhythms in humans and other species, providing significant potential for disentangling different evolutionary hypotheses for various features of rhythm.

First, we define rhythm and discuss one method for studying rhythm in vocalizations. Next, we explain why vocal rhythms are important to study, discuss the benefits of a comparative approach, and introduce several hypotheses for the evolution of different rhythm features in communication. We then synthesize evidence of rhythm in cetacean vocalizations and summarize overarching trends under a comparative lens. Finally, we discuss topics for future research. Throughout, we show how cetaceans are well positioned to address pressing questions and disentangle different evolutionary hypotheses in vocal rhythm research. Our treatment of cetacean vocalizations is meant to serve as an example of the progress that can be made by conducting comparative rhythm research at broad taxonomic scales; similar work across other taxonomic groups will be instrumental in better understanding the evolution of vocal rhythms.

## What is Rhythm?

We define rhythm as a “pattern of time intervals demarcating a sequence of stimulus events” (1). This definition requires *n* ≥ 3 “events” (here, vocalizations), *n* – 1 intervals separating those events, and some repetition for a sequence to be considered rhythmic. This broad definition can be applied across species, timescales, and event types and is compatible with an existing “definitional framework” for cross-species rhythm comparisons (2). Under this framework, series of temporal intervals can be categorized as periodic (regularly repeating) or aperiodic (nonrepeating) (*SI Appendix*, Fig. S1). All periodic signals are rhythmic under our definition and hence this paper’s focus. Periodic signals are further categorized as isochronous (metronomic: the repeated unit consists of one interval) or heterochronous (the repeated unit consists of more than one interval). For simplicity, we use the terms isochronous/isochrony and heterochronous/heterochrony to describe signals with regular behavior over any timescale.

Isochrony and heterochrony are the building blocks of rhythm. Quantifying when, where, and how often they occur in vocalizations is key to identifying promising species for vocal rhythm research and facilitating subsequent investigations of more complex rhythmic phenomena (2).

## How Can We Quantify Rhythm?

There are many methods for studying rhythm in communication (3–5). We will highlight one key metric for quantifying isochrony: the coefficient of variation (CV) of temporal intervals. Most rhythm analyses involve measuring variability in a sequence’s intervals (the durations of time between consecutive events). The CV is calculated by dividing the SD of the intervals by the mean and is expressed as a percent (*SI Appendix*, Method S1). It provides a first indication of whether a sequence is rhythmic or not: A perfectly isochronous sequence has a CV of 0%, while higher CVs can indicate either aperiodicity or heterochrony. The CV is independent of temporal scale, making it useful for comparisons across studies and species.

## Why Should We Study Rhythm?

Rhythm has been implicated in many processes and behaviors, including memory, attention, signaling, sociality, and reward (6). These links are best understood in humans. Rhythm perception in humans emerges by two months of age and continues to develop thereafter (7, 8). This “rhythm instinct” allows us to perceive, interpret, and create rhythm in music, speech, and dance (9–11). In the acoustic domain, rhythm improves our ability to detect, react to, and compare signals (12, 13). Rhythm also allows us to predict and target our attention to specific points in time (14), which ultimately allows multiple individuals to synchronize their attention and behavior (14, 15). Rhythm is thus strongly implicated in behavior production and perception in humans, but how we and other species acquired advanced rhythmic abilities is unknown.

There are numerous hypotheses on the evolutionary origins of rhythm in vocal communication (see Table 1 and *SI Appendix*, Table S1 for a selection), most of which explicitly refer to primates. These hypotheses target different features of rhythm—including isochrony, complexity, and auditory-motor entrainment [i.e., the “ability to synchronize motor output to auditory input” (16)]—and may not be mutually exclusive. While future rhythm analyses would benefit from developing more comparative hypotheses, cross-species research has already yielded insights into existing hypotheses.

**Table 1.** Cetaceans can contribute to several leading hypotheses for the evolutionary origins of features of rhythm

Hypothesis	Description	Key prediction(s)	Cetacean contribution
Vocal learning hypothesis	Vocal learning abilities are a preadaptation for rhythm production and perception abilities (17). Advanced vocal learning abilities are a preadaptation for beat perception and synchronization (BPS) (18).	Species with more advanced vocal learning abilities will have more advanced rhythm production and perception abilities.	Cetaceans are one of just eight animal groups with confirmed vocal learners (19). Odontocetes may have more advanced vocal learning abilities than mysticetes (19).
		Only species with the most advanced vocal learning abilities will be capable of BPS.	Certain odontocetes can imitate novel sounds and vocalizations from other species and should be capable of BPS (19).
Breathing hypothesis	Rhythmic capacities build upon breathing phenotypes (20–22).	Species with enhanced breathing control will have advanced vocal rhythm production abilities.	Cetaceans have extremely advanced behavioral control of breathing (23).
Sexual selection hypothesis	Rhythm, and other musical abilities, evolved due to (runaway) sexual selection for complex acoustic displays (24, 25).	Vocalizations with more rhythmic structure or complexity should be sexually selected and hence indicate increased fitness of the vocalizer and/or enhanced mate preference of the listener.	Mysticete song is likely under sexual selection and is rhythmic (4, 26), while nonsong vocalizations are not thought to be under sexual selection and seem to be less rhythmic (27, 28). Some odontocetes produce rhythmic vocalizations during courtship (29, 30), but it is unknown if these displays are under sexual selection.
Mother–infant bonding hypothesis	Rhythmic communication and entrainment evolved to establish an emotional bond during mother–infant interactions, to ensure that mothers would become committed to extended care of infants (31, 32).	Species with extended maternal care periods (and where both mothers and calves vocalize) should have more advanced vocal rhythmic abilities than those with short care periods.	Cetaceans have prolonged, but very variable, periods of calf care (33). Weaning age is later in odontocetes than mysticetes (33). Some odontocetes stay with their mothers for life (33).
		Child-directed communication (“motherese”) should be more rhythmic than communication directed at other age classes (34).	Evidence of motherese has been shown for certain mysticetes (35) and odontocetes (36).
Group display hypothesis	Individual rhythms evolved as a by-product of group displays, largely due to the need to synchronize during displays (37, 38). Synchronized group displays promote cohesion and cooperation, and also signal group quality to outsiders (37, 38).	Group-living animals will have more rhythmic communication than solitary animals.	Mysticetes have relatively simple social structures and small group sizes (39). Odontocetes typically live in groups, which fall along spectrums of size and stability (40).
		Species where individuals regularly synchronize behaviors will have advanced individual rhythm production and perception abilities versus species that rarely synchronize.	Cetaceans, particularly odontocetes, synchronize many different types of behaviors (29, 41–43). There is anecdotal evidence linking behavioral synchronization to vocal rhythms for at least one odontocete species (42).

This selection of hypotheses was chosen because cetaceans provide a natural range of related species for empirically testing key predictions. Most hypotheses come from the human literature because rhythm has been both decomposed into different features and studied most intensively in modern humans. Because some of these hypotheses target different features of rhythm, they may not be mutually exclusive. See *SI Appendix*, Table S1 for an extended version.

## What Have We Learned from Comparative Vocal Rhythm Research?

Comparative work on vocal rhythms has become increasingly common, with insights from anurans, fish, mammals, and birds. There is also substantial rhythm research on arthropods (44), which typically produce sounds via external, mechanical means (e.g., stridulation) (2). In the interests of space and scope, we do not cover that body of work here (but see ref. 45). Instead, we focus on vocal rhythms produced by vertebrates, occasionally incorporating insights from human instrumental music research as well. We define a vocalization as any internally generated acoustic sound produced by an animal, regardless of the anatomical feature(s) used to produce the sound.

Many animal vocalizations are isochronous, including those produced by anurans (46, 47), fish (48, 49), birds (50–53), rodents (54, 55), canids (56, 57), pinnipeds (58–61), primates (62, 63), and bats (64). Heterochronous vocal rhythms are seemingly rarer but have been documented in some seals (60, 61), lemurs (63), and birds (53). The prevalence of isochronous animal vocalizations suggests that isochrony is an evolutionarily basal trait of vocal communication systems, but how and why certain species learned to modify isochronous vocalizations and to develop heterochronous vocalizations is unresolved. For example, the extent to which animals can produce isochronous vocalizations at different tempi (i.e., rates) is unknown for most species but would be a useful first division for delineating species with advanced versus simple rhythmic abilities. Practically, testing whether isochrony precision is maintained as a function of tempo may point toward a rhythmic propensity beyond a simple by-product of physiological constraints (e.g., ref. 65).

Comparative research has also shown that animals often produce isochronous vocalizations in high-arousal situations. Possums (*Ailurops ursinus*) (66), dogs (*Canis familiaris*) (56, 57), fur seals (*Arctocephalus pusillus*) (58), and elephant seals (*Mirounga angustirostris*) (59) produce isochronous vocalizations at fast tempi when stressed or excited. However, rhythmicity can vary by individual (67), behavioral context (56), vocalization type (66), and background noise (68). For example, male Arabian babbler birds (*Argya squamiceps*) are highly vocal during territorial disputes, but aggressive males emit isochronous calls and lead charges, whereas timid males emit aperiodic calls and hang back (67). Male Lusitania toadfish (*Halobatrachus didactylus*) show intra- and interindividual rhythmic variability, with individuals producing both isochronous and aperiodic vocalizations (48). Dogs growl more isochronously in aggressive situations compared to play situations (56) and male blackbirds (*Turdus merula*) sing more isochronous songs during noisy dawn choruses than in the evening, when few males are singing (51). Some species also show rhythmic variation across vocalization types (66) and with background noise levels (69).

Comparative vocal rhythm research efforts have been uneven across taxa. Most studies have used nonhuman primates (hereafter primates) and songbirds as models for understanding the evolution of human rhythmic abilities, but these species force trade-offs: Primates are our closest relatives but have limited vocal abilities, while songbirds are phylogenetically distant from us but rhythmically similar (70). To understand how rhythmic abilities evolved in vertebrates generally, and in humans specifically, we must expand the breadth of species studied and the depth of analyses. Taxonomic groups that show diversity in both vocal rhythmicity and nonvocal characteristics, like cetaceans, are an ideal testbench for evolutionary hypotheses for rhythm features. As we will show, they have untapped potential to test leading evolutionary scenarios for the emergence of different features of rhythm (Table 1 and *SI Appendix*, Table S1).

## What Can Cetaceans Contribute?

The mammalian infraorder Cetacea comprises ~90 species, divided into mysticetes (baleen whales) and odontocetes (toothed whales). Compared to songbirds and primates, cetaceans are on a “sweet spot” of the aforementioned trade-offs: They are both vocal and social; are phylogenetically closer to humans than songbirds; and possess a mammalian brain, allowing us to better evaluate common ancestry and convergent evolution as drivers of rhythmic similarities (71). Cetaceans also share several traits with humans that are rare in primates, such as vocal production learning—the ability to acquire new vocalizations or modify existing ones based on experience (see “vocal learning hypothesis,” Table 1 and *SI Appendix*, Table S1) (19). Mysticetes and odontocetes also differ in key vocal and social ways, which can help address different questions about the evolution and function of vocal rhythms.

### Cetacean Vocal Behavior.

In marine environments, sound travels much faster and further than other signaling cues (e.g., visual, chemical) (72), making acoustics the primary sensory modality cetaceans use throughout life (73). Mysticetes and odontocetes differ in their sound production anatomies and generate vocalizations with very different spectro-temporal features (see “breathing hypothesis,” Table 1 and *SI Appendix*, Table S1).

Like most other mammals, mysticetes vocalize by vibrating the folds of the larynx (73). Single vocalizations generally have a low fundamental frequency (~7 to 20 Hz), contain harmonics, and last on the order of seconds (73). Many species combine single vocalizations into long, patterned, repetitive displays called “songs” (74). These songs can be hierarchically structured, with “units” (i.e., individual calls) repeated in “phrases,” phrases assembled into “themes,” and themes combined into songs (74, 75). Sexed singers have been male, suggesting that mysticete song (like birdsong) plays a role in reproduction and courtship (see “sexual selection hypothesis,” Table 1 and *SI Appendix*, Table S1) (76). In some species, like humpback whales (*Megaptera novaeangliae*), songs evolve over time via social learning (77). Mysticetes also produce “nonsong vocalizations,” like social sounds (78) and foraging calls (79), but these have been studied less.

Odontocetes vocalize by forcing air through a muscular structure in the head called the phonic lips (80). Odontocete vocalizations are typically higher frequency than mysticete vocalizations and can be divided into three broad categories: clicks, pulsed sounds, and whistles (73). Clicks are broadband (frequency varies by species), short duration, and impulsive sounds that are often used for echolocation (73). Pulsed sounds are series of broadband pulses with extremely short interpulse intervals, and whistles are narrowband, frequency-modulated signals that typically last less than a second (73). Unlike mysticetes, odontocetes do not produce songs but do still make vocalizations linked to reproduction/courtship (29, 30).

Compared to many terrestrial mammals, including humans, the Cetacea infraorder thus includes species with anatomically homologous (i.e., mysticetes) and analogous (i.e., odontocetes) ways of producing vocalizations, which bolsters their comparative potential. That potential is further enhanced by the diverse social behaviors seen in both mysticetes and odontocetes.

### Cetacean Social Behavior.

A core unit of cetacean sociality across species is the mother–calf pair. Both mysticetes and odontocetes have prolonged calf care, and many behaviors (including vocal repertoires) are socially transmitted from mother to offspring (see “mother–infant bonding hypothesis,” Table 1 and *SI Appendix*, Table S1) (81). Such socially transmitted behaviors constitute a form of culture—information or behavior that is shared within a community and acquired through social learning (82). For several species, culture is a significant driver of social behavior, group divisions, and social structure (82).

Cetacean societies are structured in many ways (see “group display hypothesis,” Table 1 and *SI Appendix*, Table S1). Group size ranges from a few to thousands of individuals, with mysticetes and odontocetes often at opposite ends of the scale (39, 40). Some odontocetes, such as sperm whales (*Physeter macrocephalus*), bottlenose dolphins (*Tursiops* spp.), and orcas (*Orcinus orca*), have hierarchically organized social structures or alliance systems (40). Cetaceans often maintain their social bonds and structures through behavioral synchronization, which occurs in various contexts and time scales (41). The mechanisms facilitating behavioral synchronization in cetaceans are rarely known.

### Our Approach.

Collectively, these vocal, social, and life history traits make cetaceans an effective group for disentangling different evolutionary hypotheses of features of rhythm (Table 1 and *SI Appendix*, Table S1). In the following sections, we illustrate this by synthesizing the state of knowledge on vocal rhythms in mysticetes and odontocetes. For each species with positive evidence, we use the definitional framework (*SI Appendix*, Fig. S1) to summarize vocal rhythms (Table 2 and *SI Appendix*, Table S2). When possible, we also calculate interval CVs as a measure of rhythmicity (*SI Appendix*, Method S1 and Table S3). The examples we provide are comprehensive but not exhaustive and our goal is twofold: 1) to show what cetaceans can contribute to our understanding of vocal rhythms and 2) to illustrate an approach that researchers studying different taxonomic groups can use to facilitate comparative rhythm research.

**Table 2.** Evidence of isochronous (I) and heterochronous (H) rhythm in mysticete (top) and odontocete (bottom) vocalizations

Family	Common name	Vocalization	Behavioral context	Rhythm summary	Selected references
Balaenopteridae (Rorquals)	Bryde’s whale	Be6 calls	Unknown	I	(83)
	Sei whale	Song	Courtship	I, H	(84)
	Omura’s whale	Song*	Courtship	I	(85)
	Blue whale	Song*	Courtship	I, H	(27, 86, 87)
	Fin whale	Song	Courtship	I, H	(88–90)
	Humpback whale	Cries	Foraging	I	(79)
		Song*	Courtship	I, H	(4, 91, 92)
	Minke whale (dwarf subspecies)	Song*	Courtship, spacing	I, H	(28, 93)
	Minke whale (Northeast Pacific subspecies)	Song	Courtship	I	(94)
Balaenidae	North Pacific right whale	Gunshots*	Unknown	I	(95)
		Song*	Courtship	I, H	(96)
	North Atlantic right whale	Screams*	Mating	I	(97)
	Bowhead whale	Song*	Courtship	I	(75, 98)
Delphinidae (Oceanic dolphins)	Atlantic spotted dolphin	Screams*	Aggression	I	(42)
		Squawks*	Aggression	I	
	Indo-Pacific bottlenose dolphin	Signature whistles*	Socializing	I	(99)
		Pop trains*	Courtship	I	(29)
	Common bottlenose dolphin	Bray/buzz bouts*	Aggression, courtship	I	(42)
		Bray-calls*	Foraging, socializing	I, H	(100, 101)
		Buzz bouts*	Aggression, courtship	I	(42)
		Signature whistles*	Socializing	I	(102)
		Whistle/buzz bouts*	Aggression, courtship	I, H	(42)
	Long-finned pilot whale	Repeated call sequences	Socializing	I	(103, 104)
	Northern right whale dolphin	Burst-pulses*	Unknown	I, H	(105)
	Orca	Discrete calls	Socializing	I	(106)
		Ultrasonic whistles	Unknown	I	(107)
Monodontidae	Narwhal	Pulsed calls*	Unknown	I, H	(108)
	Beluga whale	Echolocation	Foraging	I	(109)
Ziphiidae (Beaked whales)	Blainville’s beaked whale	Echolocation*	Foraging	I	(110)
	Northern bottlenose whale	Echolocation*	Foraging	I	(111)
	Cuvier’s beaked whale	Echolocation*	Foraging	I	(112, 113)
Physeteridae	Sperm whale	Codas	Socializing	I, H	(114, 115)
		Echolocation*	Foraging	I	(5)
		Surface clicks*	Courtship, advertising	I	(30)

**Fig. 1.**
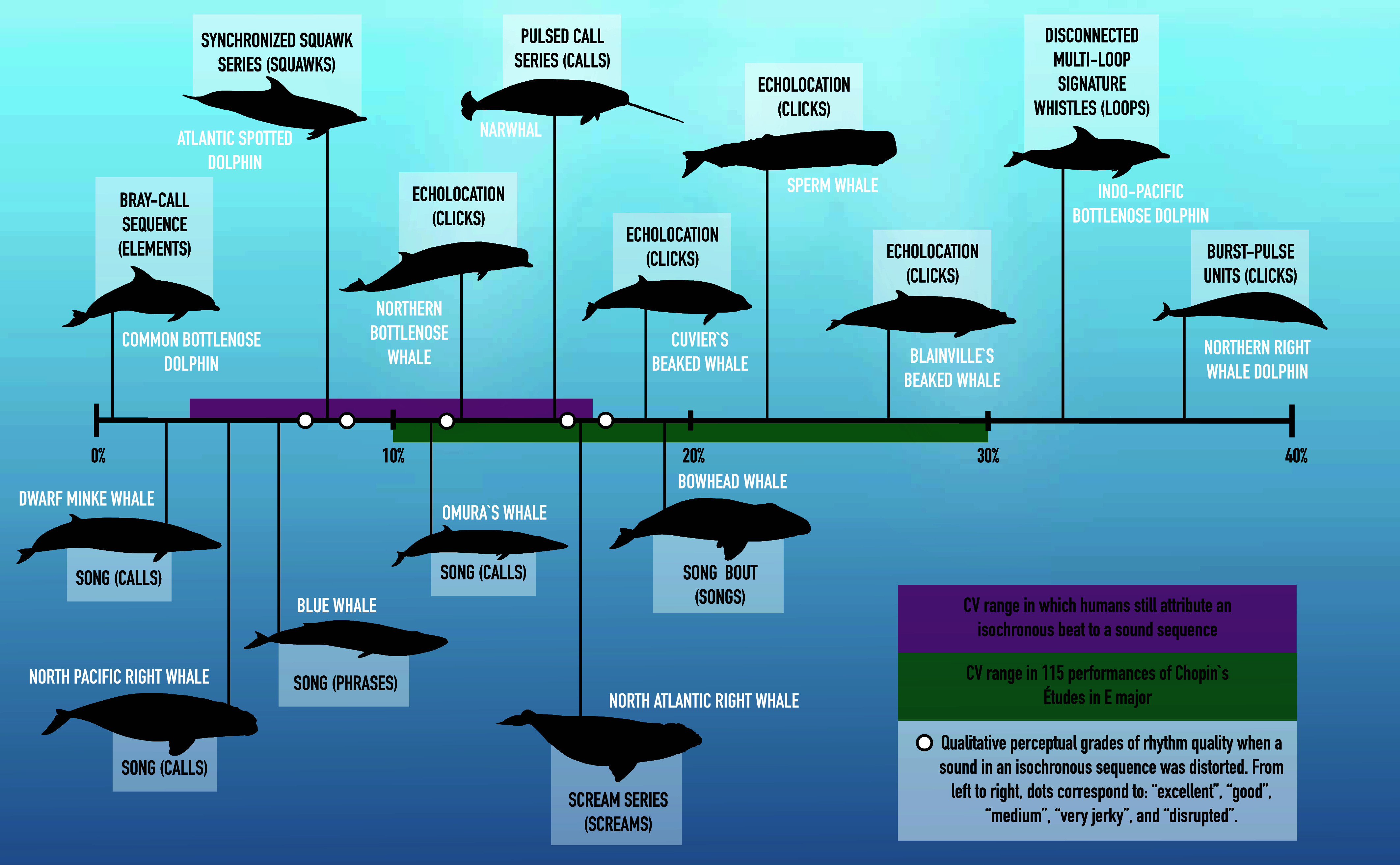
A selection of interval CV values calculated for isochronous odontocete (*Top*) and mysticete (*Bottom*) vocalizations. For each species, the vocalization type is named and the “event” for which we calculated the CV is in parentheses. We chose very isochronous vocalizations with low CVs to emphasize how precise cetacean vocal rhythms can be. References and CVs for additional vocalizations can be found in *SI Appendix*, Table S3. Colored “guideposts” from human psychophysical work (118–120) are described in the *Inset*. Cetacean images were created by Gabriel Fraga da Fonseca.

Species are arranged by phylogenetic relatedness (116). Stars denote vocalizations for which we could calculate interval CVs (*SI Appendix*, Table S3), some of which are featured in Fig. 1. See *SI Appendix*, Table S2 for full rhythm descriptions using the definitional framework (2).

## Rhythm in Cetacean Vocalizations

### Mysticetes.

Most evidence for rhythmic vocalizations in mysticetes comes from mating contexts, with at least nine (sub)species producing isochronous songs (Fig. 1, Table 2, and *SI Appendix*, Table S2). Isochronous rhythm also characterizes North Atlantic right whale (*Eubalaena glacialis*) “scream calls,” which are produced by females in mixed-sex groups and likely relate to mating (97). Both sexes of North Pacific right whales (*Eubalaena japonica*) produce broadband “gunshot” calls associated with mating, but while females produce single or few gunshots, males produce isochronous bouts of gunshots (95, 117).

Heterochrony is not as well documented but is present in the songs of at least six mysticetes: blue (*Balaenoptera musculus*) (27, 86, 87), fin (*Balaenoptera physalus*) (88), humpback (4, 91), dwarf minke (*Balaenoptera acutorostrata*) (28), North Pacific right (96), and sei (*Balaenoptera borealis*) (84) whales. It typically manifests as two or three distinct, repeated intervals within single songs (Table 2 and *SI Appendix*, Table S2). For example, in fin whale “doublet” songs, pulsed calls are separated by alternating short and long silences (*SI Appendix*, Fig. S1) (88).

Rhythm seems to be a ubiquitous feature of mysticete song. Nonsong vocalizations are comparatively understudied, but there is preliminary evidence that rhythm is less common in them. Dwarf minke whale song is rhythmic but social sounds are not (28). Similarly, blue whales produce pulsed “A” calls followed by tonal “B” calls (i.e., A-B call pairs) in both song and nonsong vocalizations, but intervals between song A-B call pairs are more isochronous (CV = 30.2%) than nonsong A-B call pairs (CV = 75.6%) (27).

### Odontocetes.

There are many examples of rhythm in odontocete vocalizations (Fig. 1, Table 2, and *SI Appendix*, Table S2). In contrast to mysticetes, these examples extend beyond courtship vocalizations (29, 30) to include vocalizations produced when foraging, socializing, and fighting.

Odontocetes use echolocation to find and track prey (73). While individuals can flexibly vary their interclick intervals based on factors like where they are in their dive cycle, distance to prey, and prey behavior (121), echolocation click trains as a whole are generally isochronous and meet our definition of rhythm (Fig. 1, Table 2, and *SI Appendix*, Fig. S1 and Table S2).

Isochronous patterns also occur within and between calls with social or affiliative functions. Bottlenose dolphins produce “signature whistles” that convey individual identity (99, 102). The spacing between repeated sound elements within signature whistles—called “loops”—is often isochronous and the interloop interval CV is smaller than the interwhistle interval CV (e.g., 21.1% vs. 124.6% in common bottlenose dolphins (*Tursiops truncatus*), averaged across 16 individuals) (99, 102). When socializing, long-finned pilot whales (*Globicephala melas*) produce “repeated call sequences” of isochronous calls (103, 104).

Rhythmic vocalizations are also produced during aggressive interactions among common bottlenose dolphins and Atlantic spotted dolphins (*Stenella frontalis*) (42). In such interactions, many different types of vocalizations, including “screams,” “squawks,” “brays,” and “buzzes,” form isochronous series (42). Common bottlenose dolphins also produce heterochronous bouts of whistles and buzzes in aggressive and courtship contexts (42).

Heterochronous rhythm characterizes the vocalizations of at least three additional species. Northern right whale dolphins (*Lissodelphis borealis*) produce at least eight types of “burst-pulse series”, all of which are heterochronous but have unknown function (105). Narwhals (*Monodon monoceros*) produce both isochronous and heterochronous series of pulsed calls, which also have unknown function (122). When socializing, sperm whales produce isochronous and heterochronous “codas” (stereotyped patterns of clicks), which are repeated in isochronous “coda bouts” (114, 115). Certain sperm whale cultural “clans” preferentially produce specific rhythmic patterns in their codas, with some clans favoring isochronous codas while others favor heterochronous codas (114).

## Overarching Trends

The trends in mysticete and odontocete vocalizations support four new conclusions:

1. Rhythm is a common feature of cetacean vocalizations.
2. Isochrony is more commonly documented than heterochrony in cetacean vocalizations.
3. Rhythm in mysticetes is largely restricted to song.
4. Rhythm in odontocetes occurs across diverse vocalization types.

We discuss each conclusion below, considering cross-species research.

### Rhythm Is Common.

We find quantitative evidence of rhythm in 34 vocalizations from 23 cetacean (sub)species (Table 2 and *SI Appendix*, Table S2). This likely underestimates the actual diversity, given that few studies have explicitly quantified rhythm in cetacean vocalizations. The rhythmic patterning we document could be a biomechanical artifact of physiological sound production constraints, as is often the case in anurans (46), or it could indicate higher-level mental processes (53). To tease these apart, researchers must consider sound production anatomy in tandem with ecological and behavioral correlates in future vocal rhythm research (53).

Why is rhythm so common in cetacean vocalizations? One factor could be the marine environment. Environmental conditions can drive trade-offs between temporal and spectral resolution in vocalizations and affect the prevalence of rhythm (3, 68). Temporal features of vocalizations are generally more resilient to propagation effects and transmission loss than spectral features (123). Producing rhythmic vocalizations is one way of improving signal transmission in noisy environments (124), and studies on fish suggest that rhythm is useful for encoding information in marine environments (69, 123).

From cross-species research, we also know that rhythm plays important roles in memory and attention. When acoustic stimuli are isochronous (versus aperiodic), humans are better at detecting, reacting to, and comparing target sounds (12, 13), and young zebra finches (*Taeniopygia guttata*) learn new songs more accurately (125). Whether these links between rhythm production and perception are widespread or restricted to a few species is unknown, but targeted vocal rhythm research on cetaceans and other taxa can help address this knowledge gap. There is neurobiological evidence linking attention and rhythm in common bottlenose dolphins: Individuals dampen their hearing sensitivity when acoustically warned about upcoming loud sounds, but the duration and extent of this dampening is lessened when loud sounds occur predictably (i.e., isochronously) after warning sounds (126). Isochrony thus lets common bottlenose dolphins precisely pinpoint when to dampen their hearing (126). Whether rhythm allows other cetaceans to effectively modulate attention could be tested by adapting the so-called “oddball paradigm” from human cognitive neuroscience to acoustic playback experiments: Individuals could be exposed to sequences of rhythmic sounds which do or do not violate temporal expectations, and their behavioral response could be measured and compared across conditions (127).

### Isochrony Is more Common than Heterochrony.

We document more examples of isochronous cetacean vocalizations (n = 34) than heterochronous ones (n = 11) (Table 2 and *SI Appendix*, Table S2), which mirrors patterns in other taxa. From a signal processing perspective, isochronous signals are powerful because they are deterministic, predictable, and energetically cheap (37, 128). Through isochronous repetition, a signaler can minimize signal entropy, maximize signal redundancy, and generate temporal expectations in listeners (74, 128). While heterochronous signals can generate expectations too, simple rhythms are easier to track, encode, and synchronize to than complex rhythms, at least in humans (8, 129, 130). Isochrony may itself be a by-product of the fundamental need to synchronize behavior, given that isochronous rhythms make the timing of upcoming signals predictable to multiple individuals (6, 37, 131, 132).

Behavioral synchronization is common in cetaceans (41) and could explain the prevalence of isochrony in cetacean vocalizations. For example, Atlantic spotted dolphins produce “squawks” during aggressive interactions; anecdotally, squawk production becomes more isochronous as multiple individuals’ body movements become more synchronized, but it is difficult to determine causality in this case study without further investigation (42). In humans, rhythm can facilitate synchronization and its numerous prosocial benefits, including increasing feelings of trust and willingness to help between partners (6, 133). Positive feelings among in-group members could benefit cetaceans as well (41).

If isochronous vocalizations are so powerful, why would heterochronous vocalizations ever evolve? One possible driver is a need for expressivity—the capacity to convey different meanings—in communication systems (128). While isochrony optimizes signal transmission fidelity and predictability, it leaves little room for expressivity. Heterochronous signals may have evolved to enable expressivity in the temporal domain.

### Rhythm in Mysticetes Is Typical of Song.

Rhythm seems to be more abundant in mysticete song compared to nonsong vocalizations, perhaps because definitions of song often include rhythm as a diagnostic feature (e.g., ref. (96)). Songs are also much more studied than nonsong vocalizations. As research on nonsong vocalizations increases (43), examples of rhythm may increase as well. Given evidence from other taxa (discussed below), however, we posit that a rhythmic disparity between mysticete song and nonsong vocalizations may persist even after accounting for research effort due to differences in vocalization complexity, function, and/or scale.

For most mysticetes, songs are their most complex vocalizations and may be more challenging to remember or produce than nonsong vocalizations. This is best emphasized for species with hierarchically structured and rapidly changing songs, like humpback and bowhead (*Balaena mysticetus*) whales (74, 77, 134). Researchers have hypothesized that rhythm acts as a mnemonic device in humpback whale song, helping males learn and remember the complex and annually evolving vocal displays (91, 92, 135). Quantitative analyses support this: Rhythmically predictable themes are the most abundant theme type in humpback songs, and song parts that are most likely to change from year to year are also the most rhythmic (92). In bowhead whales, “complex songs” made up of many call types are more stereotyped and repetitive than “simple songs” made up of one call type, providing another link between rhythm and song complexity (98). These examples mirror Australian pied butcherbirds (*Cracticus nigrogularis*), where song repertoire complexity is correlated with singing rhythmicity: Males who sing songs with more phrase types also sing more rhythmically (52). Singing mysticetes could similarly benefit from the memory-enhancing capabilities of rhythm (6, 26, 52, 125).

Vocal rhythms can guide listener attention to important signal features and have been linked to reproductive success in several species. Male rock hyraxes (*Procavia capensis*) that maintain an isochronous song rhythm have higher reproductive success (136), and male great tits (*Parus major*) that produce more rhythmic songs with more syllables have more offspring (137). Additionally, male Lusitania toadfish with better body condition can sustain higher isochronous calling rates (48). Female zebra finches may use the isochronous rhythm in males’ songs to “tune in” and assess male consistency throughout a performance (138). Vocal rhythms can thus indicate male quality by advertising singer stamina, physical coordination, or creative ability (16, 139). This could lead to sexual selection on vocal rhythms in courtship displays, like mysticete song. This seems to be the case in anurans, where vocalization timing significantly influences mate choice and temporal features of male vocalizations are under strong sexual selection (140, 141). Thus, the hypothesized function of mysticete song—as a courtship display used to secure mating opportunities—could lend itself to rhythm, whereas the different functions of nonsong vocalizations might not.

Differences in scale between song and nonsong vocalizations could also generate differences in rhythm prevalence. Mysticete song is primarily a long-range communication signal, whereas nonsong vocalizations are often made in close aggregations of individuals (43). Proximity to other animals may render some of the spatial benefits of vocal rhythm less useful or necessary in nonsong vocalizations (47, 142). For example, vocal rhythms can modulate interactions of dispersed individuals by providing information on a signaler’s location or identity (67, 89, 142). Dwarf minke whales use rhythm to maintain spacing among multiple singers, with individuals increasing their song tempo and moving away when other singers approach (28, 93). This likely reduces physical conflicts among singers competing for female attention. Vocalizations also regulate male spacing in several anurans; for example, Pacific treefrogs (*Pseudacris regilla*) produce isochronous “encounter calls” when other males start calling nearby, which discourage intruders from continuing to approach (47). Finally, because short-distance vocalizations are likely less degraded by the environment, rhythm may offer less of a benefit (3).

### Rhythm Occurs across Odontocete Vocalizations.

In contrast to mysticetes, rhythm is prevalent across different vocalization types in odontocetes. We hypothesize that the evolution of odontocete echolocation and corresponding high-resolution auditory system contributed to this prevalence.

Odontocete echolocation evolved ~28 Mya to exploit untapped food niches (143). Given that sound travels quickly in seawater—about 1,500 m/s, over four times faster than in air (72)—marine echolocators need an auditory system with high temporal resolution to rapidly interpret echoes returning from their clicks. Indeed, the temporal resolution of the odontocete auditory system exceeds that of most mammals, with clear evidence that odontocete brains respond isochronously to isochronous stimuli and can track rapid series of clicks (144). The echolocation process may lend itself to isochrony, as a result of hitting the upper limit of high-speed click production while still being able to discriminate returning echoes (145). Similar rhythmic strategies to prevent information in vocalizations from being masked may underpin the isochronous timing observed in, for example, sperm whale antiphonal coda exchanges (115).

The anatomical and neural substrates for echolocation likely underpin rhythm in other types of odontocete vocalizations. Three of the best-studied odontocetes—common bottlenose dolphins, Indo-Pacific bottlenose dolphins (*Tursiops aduncus*), and sperm whales—all show isochronous rhythm in vocalizations across behavioral contexts (Table 2 and *SI Appendix*, Table S2): For these species, rhythm is a fundamental feature of communication. This mirrors another echolocating species, the greater sac-winged bat (*Saccopteryx bilineata*), which produces isochronous echolocation calls, male territorial songs, and pup calls (64). While the acoustic properties and range of echolocation vary significantly when comparing bats to odontocetes (146), this convergence suggests that some rhythmic faculties, originally evolved for echolocation, may have been exapted for communication—including conveying various levels of individual or group identity and facilitating behavioral synchronization (29, 41, 42, 142). This hypothesis could be tested by building and comparing two “rhythmic phylogenies,” one for echolocation rhythms and the other for communicative rhythms (e.g., ref. 147).

## Outstanding Questions and Future Directions

Much of what we learned about cetacean vocal rhythms came from studies that incidentally reported temporal features of vocalizations, but dedicated studies are the best way to conclusively show whether a given species produces rhythmic vocalizations. Additionally, while we have focused on positive evidence, negative evidence is essential for mapping the evolution of traits, including vocal rhythms (e.g., ref. 49). A recent rhythm reporting framework (148) can help animal communication researchers quantify and share both positive and negative vocal rhythm results. Some promising avenues for future research include the following:

1. *Rhythm in hierarchical vocal displays:* Most analyses of temporal structure focus only on the most granular level, even though many species produce hierarchically organized vocal displays. For mysticetes with hierarchical songs, like humpback (74), bowhead (98), North Pacific right (96), and sei (84) whales, intervals are often measured at the “call” level but rarely at higher “phrase” or “song” levels. Traditional analyses accordingly fail to capture the full rhythmic complexity of these displays (62, 63). Several odontocetes also produce nested rhythmic vocal patterns. For example, Indo-Pacific bottlenose dolphins produce isochronous pops in trains and isochronous trains in sequences (29). Recent analyses of a similar phenomenon in male orangutan (*Pongo pygmaeus wurmbii*) calls provided evidence of recursive vocal motifs, which are largely unknown outside human music (62). Hierarchical analyses of Australian pied butcherbird songs found that while each song level (i.e., notes, phrases, bouts) is rhythmic, higher levels of song structure are more isochronous than lower levels (52). Future research should quantify rhythm at all levels of hierarchical vocal displays, because understanding such “rhythmic hierarchies” may inform us about cognitive and neural traits underlying information packaging in species where experimental or neural approaches are not feasible or available (63).
2. *Heterochrony as a means to expressivity:* We hypothesize that heterochronous rhythms evolved to enable expressivity in the temporal domain of vocal signals. If true, we predict that heterochrony will be more common in signals that convey identity compared to, for example, foraging calls, because multiple identities require distinct signals. This could be examined by comparing rhythmic complexity across different types of vocalizations in species that produce individual (e.g., olive frogs, *Babina adenopleura*, ref. 149) or group (e.g., long-billed hermit birds, *Phaethornis longirostris*, ref. 150) identity signals. Recent work on sperm whales found evidence that certain coda types with distinct, often heterochronous rhythmic patterns act as symbolic markers of cultural group identity (151). These heterochronous codas contrast with other isochronous sperm whale vocalizations (Table 2 and *SI Appendix*, Table S2), providing preliminary support for our hypothesis.
3. *Entrainment and multimodal rhythms:* Many evolutionary hypotheses on rhythm invoke a crucial step of developing auditory-motor entrainment. It is unknown whether any cetaceans are capable of spontaneous entrainment to externally generated acoustic rhythms, but this ability is foundational to the “vocal learning hypothesis” (Table 1 and *SI Appendix*, Table S1) and would be useful to test in cetaceans. Furthermore, rhythm is multimodal, with neural, bodily, and interactive rhythms all contributing to the communicative rhythms of animals (152). For example, bats often couple wingbeats to echolocation and respiration during flight (64, 142). Whether other echolocators, like odontocetes or shrews (153), also couple echolocation with bodily movements or breathing to generate multimodal rhythms is unknown. Sound and movement recording tags (154) can be used to determine whether cetaceans are capable of bodily entrainment to external acoustic rhythms and whether echolocation is linked to rhythms in other modalities in odontocetes.
4. *Measuring vocal complexity as rhythmic complexity:* There are established correlations between social and vocal complexity across taxa (155), but vocal complexity is rarely measured in terms of rhythmic complexity. However, hierarchical temporal structure—one measure of rhythmic complexity—in human and animal vocalizations is enhanced by social interactions (156). For example, orca vocalizations used during interactions have more hierarchical temporal structure (on par with human conversations) than songs from solitary male humpback whales (156). This suggests that social complexity and vocal rhythmic complexity are correlated or coevolved traits, and the abundance of rhythmic odontocete vocalizations may reflect their generally more complex societies compared to mysticetes. Given these patterns, we urge work on the interplay between social and vocal complexity to include measures of rhythmic complexity.
5. *Rhythm and culture:* In humans, culture has known interactions with rhythm production, transmission, and perception (157–159). For example, musical rhythms are typically distributed categorically around small-integer ratios (e.g., 1:1, 1:2, 3:1) and different cultures exhibit biases toward producing different ratios (50, 158). Rock hyraxes (136), songbirds (50), and primates (63) also produce vocal rhythms with small-integer ratios, but it is unknown whether different cultural groups of animals exhibit biases toward different ratios, as human cultures do. Cetaceans are an excellent model for investigating culture/rhythm interactions, given that some species are multicultural and produce rhythmic vocalizations (82). These species can help test outstanding hypotheses, including that categorical rhythms promote or emerge from cultural transmission of learned vocalizations (50).

## Conclusions

Vocal rhythms can significantly augment animal behavior and interactions, but most rhythm research has focused on a handful of taxa. To answer big-picture vocal rhythm questions, we must broaden the species studied and deepen our analyses. As we have argued here, cetaceans are a fruitful next frontier for vocal rhythm research. Our synthesis shows that cetaceans not only frequently produce rhythmic vocalizations but also exhibit behaviors known or hypothesized to drive rhythm production in other taxa. By explicitly quantifying rhythm in cetacean vocalizations and inducting additional taxa into the comparative approach for vocal rhythm research, we can better disentangle different hypotheses for the evolution of rhythm in communication systems.

## Supplementary Material

Appendix 01 (PDF)

## Data Availability

All other data are included in the manuscript and/or *SI Appendix*.
