# Supplementary material for: Cetaceans are the next frontier for vocal rhythm research
Source: Proc Natl Acad Sci U S A. 2024 May 30;121(25):e2313093121. doi: 10.1073/pnas.2313093121 (PMC11194516; doi:10.1073/pnas.2313093121)
Supplement: Supplementary file 1 — Appendix 01 (PDF) [file pnas.2313093121.sapp.pdf]

PNAS

Supporting Information for

Cetaceans are the next frontier for vocal rhythm research.

Taylor A. Hersh, Andrea Ravignani, Hal Whitehead

Corresponding author: Taylor A. Hersh
Email: taylor.a.hersh@gmail.com

This PDF file includes:

Method S1
Figure S1
Tables S1–S3
Supplemental References

Method S1. Calculating interval CVs from parameters reported in the cetacean literature. Few studies have explicitly focused on quantifying rhythm in cetacean vocalizations, but some provide metrics that we can retroactively use to do so. Most cetacean papers that measure temporal features of vocalizations present summary statistics for inter-event intervals (IEIs; the durations of *silences* separating consecutive events) rather than inter-onset intervals (IOIs; the durations of time between the *starts* of consecutive events). This is not necessarily an issue: silences can be just as or more important than sounds in rhythm production and perceptions (1–3); IEIs and IOIs are highly correlated if event duration is relatively consistent; and CVs can still be calculated for IEIs to get an initial sense of rhythmic regularity. Given this precedent, our quantification of rhythm in cetacean vocalizations is generally derived from IEIs, but we recommend that cetacean researchers report IOIs in the future to foster comparability and consistency with other rhythm researchers.

On a methodological note, no universal threshold exists for how close to 0% a CV must be to be perceived as isochronous (4). Humans still characterize a sound sequence as isochronous when the sound onsets are distorted from isochrony by ~4–17% (5), but similar (and essential) psychophysical studies have not been done for most other species. Rather than setting an arbitrary and likely inappropriate CV threshold for isochrony for non-human animal vocalizations, we propose considering species- and vocalization-specific CV values along a “more-to-less-isochronous” continuum (Figure 2), with several human-derived metrics (5–7) as guideposts. As additional psychophysical work is done on non-human animals, these guideposts should be updated to include other species.

As mentioned in the main text, we are interested in rhythm over any timescale. Given the average duration of cetacean vocalizations, timescales are typically on the order of seconds to minutes for mysticetes and milliseconds to seconds for odontocetes.

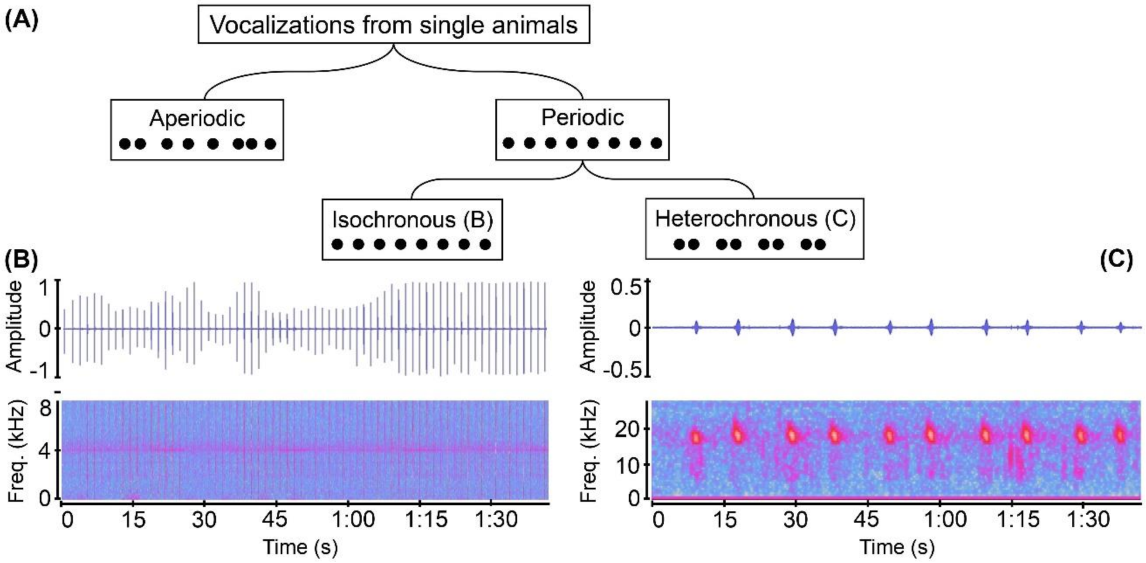

Figure S1. Definitional framework for characterizing vocal rhythms, adapted from (8). (A) The temporal structure of vocalizations can be described using this decision tree. Visual examples depict the timing of a sequence of vocalizations (black dots). Waveforms (top) and spectrograms (bottom) show (B) an isochronous sequence of sperm whale echolocation clicks (inter-click intervals=1.48±0.04 s, CV=2.7%) (9) and (C) a heterochronous sequence of pulses in fin whale song (10). In (C), short (8.55±0.24 s, CV=2.8%) and long (11.34±0.09 s, CV=0.8%) inter-pulse intervals alternate.

Table S1. Extended version of Table 1 with added details in italics. See Table 1 caption for details.

Hypothesis	*Description*	*Key prediction(s)*	*Cetacean contribution*
Vocal learning hypothesis	Vocal learning abilities are a preadaptation for rhythm production and perception abilities (11). Advanced vocal learning abilities are a preadaptation *for a specific form of rhythmic entrainment: the ability to spontaneously perceive a beat and synchronize bodily movements to it* (i.e., beat perception and synchronization, BPS) (12).	Species with more advanced vocal learning abilities will have more advanced rhythm production and perception abilities.	Cetaceans are one of just eight animal groups with confirmed vocal learners, *and both mysticetes (e.g., humpback whales, bowhead whales) and odontocetes (e.g., orcas, beluga whales, Risso's dolphins, bottlenose dolphins) are represented* (13). Odontocetes may have more advanced vocal learning abilities than mysticetes, *and are typically grouped with humans at the pinnacle of such abilities* (13).
		Only species with the most advanced vocal learning abilities *(e.g., the ability to imitate novel sounds or the vocalizations of other species (13)) will be able to spontaneously perceive and synchronize to externally generated acoustic rhythms* (i.e., be capable of BPS).	Certain odontocetes can imitate novel sounds and vocalizations from other species (*this ability has not yet been recorded in mysticetes*) and should be capable of BPS (13).
Breathing hypothesis	Rhythmic capacities—*in particular, isochrony and meter*—build upon breathing phenotypes (14–16).	Species with enhanced breathing control will have advanced vocal rhythm production abilities. *For example, they will be better able to produce and imitate vocal rhythmic patterns than species with limited breathing control, as breathing and vocalizing typically both rely on breath control.*	*As conscious breathers,* cetaceans have extremely advanced behavioral control of breathing (17). *A spectrum of abilities also exists, with significant inter-specific variation in breathing anatomy, function, and capacity (17). Odontocetes are generally more extreme in behaviors related to breathing (e.g., dive depth, dive duration, swimming speed) than mysticetes, and may thus have more advanced breathing control (17). Unlike other species for which this hypothesis has been considered, however, mysticetes and odontocetes are capable of recirculating air and can produce many vocalizations on a single breath* (18)*. Cetaceans could thus be a counterexample to the key prediction of this hypothesis, given that vocalizing and breathing are disconnected in cetaceans in a way rarely seen among mammals* (18)*.*
Sexual selection hypothesis	Rhythm, and other musical abilities, evolved due to (runaway) sexual selection for complex acoustic displays (19, 20).	Vocalizations with more rhythmic structure or complexity should be sexually selected and hence indicate increased fitness of the vocalizer and/or enhanced mate preference of the listener.	Mysticete song is likely under sexual selection and is rhythmic (21, 22), while non-song vocalizations are not thought to be under sexual selection and seem to be less rhythmic (23, 24). *Quantifying song rhythmic structure and complexity and comparing it with various measures of male reproductive success (e.g., number of mating opportunities, length of consortships, number of paternities) across individuals could indicate whether vocal rhythm specifically is under sexual selection in mysticetes.* Some odontocetes produce rhythmic vocalizations during courtship (25, 26), but it is unknown if these displays are under sexual selection; *similar analyses comparing vocalization rhythmicity and reproductive success could be done for those species.*
Mother-infant bonding hypothesis	Rhythmic communication and entrainment evolved to establish an emotional bond during mother-infant	Species with extended maternal care periods (and where both mothers and calves vocalize) should have more	Cetaceans have prolonged, but very variable, periods of calf care (29). Weaning age is later in odontocetes *(~16.5 months)* than mysticetes *(~7 months)* (29). *Post-weaning maternal care is limited in mysticetes,* while some odontocetes stay with their mothers for life (29).

	interactions, to ensure that mothers would become committed to extended care of infants (27, 28).	advanced vocal rhythmic abilities than those with short care periods.	
		Child-directed communication ("motherese") should be more rhythmic than communication directed at other age classes (30).	Evidence of motherese has been shown for certain mysticetes (31) and odontocetes (32). *Such evidence has manifested as vocalizations specific to mother-calf contexts (e.g., grey whales) or spectrally-modified vocalizations (e.g., common bottlenose dolphins)* (31, 32)*. Very few studies have specifically investigated motherese in the form of rhythmic/temporal modifications to vocalizations, although a recent study found that common bottlenose dolphin mothers altered spectral, but not temporal, features of whistles in the presence of their calves* (32)*.*
Group display hypothesis	Individual rhythms—*in particular, isochrony*—evolved as a byproduct of group displays, largely due to the need to synchronize during displays (33, 34). Synchronized group displays promote cohesion and cooperation, and also signal group quality to outsiders (33, 34).	Group-living animals will have more rhythmic communication than solitary animals.	*While mysticetes do coalesce on shared breeding or feeding grounds,* they typically have relatively simple social structures and small group sizes *outside of the breeding season* (35). *Outside of the mother-calf dyad, most mysticetes are thus considered solitary* (35, 36)*. This contrasts with group-living odontocetes* (37)*.* Odontocetes typically live in groups, which fall along spectrums of size *(from few to thousands)* and stability *(undifferentiated relationships, weak community structure, fission-fusion networks, long-term groups, etc.)* (37).
		Species where individuals regularly synchronize behaviors will have advanced individual rhythm production and perception abilities versus species that rarely synchronize.	Cetaceans, particularly odontocetes, synchronize many different types of behaviors, *including breathing, swimming, migrating, and vocalizing* (25, 38–40). There is anecdotal evidence linking behavioral synchronization to vocal rhythms for at least one odontocete species, *the Atlantic spotted dolphin* (38)*. Indo-Pacific bottlenose dolphins in Shark Bay, Western Australia would be an interesting case study: there, males work together in long-term alliances to gain reproductive access to females* (25)*. They synchronize their behaviors and their (isochronous) vocalizations when cooperatively guarding females from rival alliances* (25)*.*

Table S2. Extended version of Table 2 with rhythm descriptions (column 5) following the definitional framework (8). See Table 2 caption for details.

Family	Common name	Vocalization	Behavioral context	Rhythm description	Rhythm summary	Selected references
Balaenopteridae (Rorquals)	Bryde's whale	Be6 calls	Unknown	Isochronous sweeps in Be6 calls; isochronous Be6 calls in series	I	(67)
	Sei whale	Song	Courtship	Isochronous and heterochronous downsweeps in songs	I, H	(68)
	Omura's whale	Song*	Courtship	Isochronous 15-50 Hz amplitude-modulated calls in songs	I	(42)
	Blue whale	Song*	Courtship	Isochronous and heterochronous calls in phrases; isochronous phrases in sequences; isochronous sequences in songs	I, H	(24, 44, 46)
	Fin whale	Song	Courtship	Isochronous and heterochronous 20 Hz pulses in songs	I, H	(69–71)
	Humpback whale	Cries	Foraging	Isochronous cries in series	I	(72)
		Song*	Courtship	Isochronous and heterochronous units in phrases; isochronous phrases in songs	I, H	(21, 48, 73)
	Minke whale (dwarf subspecies)	Song*	Courtship, spacing	Isochronous "Star Wars" vocalizations in slow and fast songs; heterochronous Star Wars vocalizations in rapid-clustered songs	I, H	(23, 49)
	Minke whale (Northeast Pacific subspecies)	Song	Courtship	Isochronous boings in songs	I	(74)
Balaenidae	North Pacific right whale	Gunshots*	Unknown	Isochronous gunshots in bouts	I	(75)
		Song*	Courtship	Isochronous and heterochronous calls in phrases; isochronous phrases in song	I, H	(51)
	North Atlantic right whale	Screams*	Mating	Isochronous screams in series	I	(52)
	Bowhead whale	Song*	Courtship	Isochronous units in songs; isochronous songs in song bouts	I	(53, 54)
Delphinidae (Oceanic dolphins)	Atlantic spotted dolphin	Screams*	Aggression	Isochronous screams in series	I	(38)
		Squawks*	Aggression	Isochronous squawks in series	I	
	Indo-Pacific bottlenose dolphin	Signature whistles*	Socializing	Isochronous loops in disconnected multi-loop signature whistles	I	(55)
		Pop trains*	Courtship	Isochronous pops in pop trains; isochronous pop trains in sequences	I	(25)
	Common bottlenose dolphin	Bray/buzz bouts*	Aggression, courtship	Isochronous bray/buzz bouts in series	I	(38)
		Bray-calls*	Foraging, socializing	Isochronous and heterochronous elements (e.g., gulps, pops, grunts, cracks, squeaks) in sequences	I, H	(56, 76)
		Buzz bouts*	Aggression, courtship	Isochronous buzzes in series	I	(38)
		Signature whistles*	Socializing	Isochronous loops in disconnected multi-loop signature whistles	I	(57)

		Whistle/buzz bouts*	Aggression, courtship	Heterochronous whistles and buzzes in bouts; isochronous bouts in series	I, H	(38)
	Long-finned pilot whale	Repeated call sequences	Socializing	Isochronous calls in sequences	I	(77, 78)
	Northern right whale dolphin	Burst-pulses*	Unknown	Isochronous clicks in burst-pulse units; heterochronous burst-pulse units in series	I, H	(58)
	Orca	Discrete calls	Socializing, social travelling	Isochronous discrete calls in series	I	(79)
		Ultrasonic whistles	Unknown	Isochronous ultrasonic whistles in series	I	(80)
Monodontidae	Narwhal	Pulsed calls*	Unknown	Isochronous and heterochronous calls in series	I, H	(59)
	Beluga whale	Echolocation	Foraging	Isochronous clicks within packets; isochronous packets within series	I	(81)
Ziphiidae (Beaked whales)	Blainville’s beaked whale	Echolocation*	Foraging	Isochronous clicks in series	I	(60)
	Northern bottlenose whale	Echolocation*	Foraging	Isochronous clicks in series	I	(61)
	Cuvier’s beaked whale	Echolocation*	Foraging	Isochronous clicks in series	I	(62, 63)
Physeteridae	Sperm whale	Codas	Socializing	Isochronous and heterochronous clicks in codas; isochronous codas in bouts	I, H	(82, 83)
		Echolocation*	Foraging	Isochronous clicks in series	I	(64)
		Surface clicks*	Courtship, advertising	Isochronous clicks in series	I	(26)

Table S3. Coefficients of variation (CVs) for examples of isochronous (I) and heterochronous (H) rhythm in mysticete (top) and odontocete (bottom) vocalizations. Species are arranged by phylogenetic relatedness (41). For family names and the behavioral context in which each vocalization is produced, see Table 2. The “Unit” column gives the acoustic unit of interest in the vocalization, and rhythm is considered at the level of the inter-unit interval. The “Reference” column gives the reference (and, when appropriate, relevant figures/tables) for the values in the “CVs” column. The CVs used in Figure 2 are bolded. We conceptualized heterochronous rhythms as multiple overlaid isochronous rhythms and calculated CVs separately for each constituent isochronous rhythm.

Common name	Vocalization	Unit	Rhythm summary	Reference	CVs
Omura’s whale	Song	15-50 Hz amplitude-modulated call	I	(42)	**Inter-call interval: 152.9±16.47 s (10.8%)**
Blue whale	Song	Call[1]	H	(44): Table 4	A-B interval: 4.6±1.79 s (38.9%)<br>B-A interval: 73.3±5.0 s (6.8%)
				(24): Table 2	A-B interval: 47.8±3.7 s (7.7%)<br>B-A interval: 83.0±25.1 s (30.2%)
				(46)	A-B interval: 25.6±6.2 s (24.2%)<br>B-B interval (rare, occurs when an additional B is present): 30.6±0.7 s (2.3%)<br>B-A interval: 58.2±6.5 s (11.2%)
		Phrase[2]	I	(44): Table 4 and text	Inter-phrase interval:<br>73.3±5.0 s (6.8%) (all sessions)<br>**75.5±4.3 s (5.7%) (single session)**
				(24): Table 2	Inter-phrase interval: 83.0±25.1 s (30.2%)
				(46)	Inter-phrase interval: 58.2±6.5 s (11.2%)
		Sequence[3]	I	(44): Table 2	Inter-sequence interval (all durations): 237.7±75.5 s (31.8%)<br>Inter-sequence interval (< 500 s): 193.6±46.0 s (23.8%)
Humpback whale	Song	Unit[4]	H	(48): Table 1	AB song phrases (for whales 1, 2, and 3, respectively):<br>A-B interval: 1.91±0.26 s (13.6%), 2.17±0.38 s (17.5%), 1.95±0.16 s (8.2%)<br>B-A interval: 0.82±0.24 s (29.3%), 0.94±0.36 s (38.3%), 0.66±0.39 s (59.1%)<br>BBCCD song phrases (for whales 1 and 2, respectively):<br>B-B interval: 1.95±0.33 s (16.9%), 2.07±0.28 s (13.5%)<br>B-C interval: 1.24±0.39 s (31.5%), 1.05±0.20 s (19.0%)<br>C-C interval: 0.87±0.44 s (50.6%), 0.86±0.30 s (34.9%)<br>C-D interval: 0.86±0.30 s (34.9%), 0.75±0.23 s (30.7%)<br>D-B interval: 2.08±0.19 s (9.1%), 2.15±0.21 s (9.8%)<br>BB’CCD song phrases (for whales 1, 2, and 3, respectively):<br>B-B’ interval: 2.21±0.12 s (5.4%), 2.37±0.37 s (15.6%), 2.10±0.23 s (11.0%)

1 Individual sound units (43); called 'parts' in (44); include call types A, B, C, D, and E (45)

2 An organized combination of calls (44); in A-only and B-only phrases, the phrase is the single A or B call, respectively; in non-contiguous A-B phrases, the phrase comprises the A call, the B call, and the intervening silent interval; in contiguous A-B phrases, the phrase comprises the combined A and B call (with no silent interval separating them)

3 "One or more phrases repeated in a regular cadence" (44)

4 Each individual sound (47)

					B'-C interval: 1.59±0.12 s (7.5%), 2.06±0.47 s (22.8%), 2.05±0.26 s (12.7%)<br>C-C interval: 1.07±0.06 s (5.6%), 1.32±0.07 s (5.3%), 1.20±0.32 s (26.7%)<br>C-D interval: 0.61±0.07 s (11.5%), 0.77±0.14 s (18.2%), 0.51±0.11 s (21.6%)<br>D-B interval: 1.80 s (n=1), 2.05±0.40 s (19.5%), 1.80±0.22 s (12.2%)
		Phrase	I	(48): Table 1	Inter-AB phrase interval (for whales 1, 2, and 3, respectively): 0.82±0.24 s (29.3%), 0.94±0.36 s (38.3%), 0.66±0.39 s (59.1%)<br>Inter-BBCCD phrase interval (for whales 1 and 2, respectively): D-B interval: 2.08±0.19 s (9.1%), 2.15±0.21 s (9.8%)<br>Inter-BB'CCD phrase interval (for whales 2, and 3, respectively): 2.05±0.40 s (19.5%), 1.80±0.22 s (12.2%)
Minke whale (dwarf subspecies)	Song	Star wars vocalization	I	(49)	Inter-star wars vocalization intervals for three fast songs[5]: 32.0±1.6 (5.0%), 33.2±1.7 s (5.1%), and 32.9±2.2 s (6.7%)
			I	(23): Table 3.2 and text	Inter-star wars vocalization interval (slow song[6]): 251.7±50.0 s (19.9%)<br>(CVs per individual (n=20) ranged from 4.3% to 32.9%)
			I	(23): Table 3.2 and text	Inter-star wars vocalization interval (fast song): 32.7±2.6 s (8.0%)<br>(CVs per individual (n=18) ranged from **2.2%** to 11.2%)
			H	(23): Table 3.2 and text	Inter-star wars vocalization interval 1 (rapid-clustered song[7]): 4.7±1.5 s (31.9%)<br>(CVs per individual (n=8) ranged from 4.8% to 86.2%)<br>Inter-star wars vocalization interval 2 (rapid-clustered song): 17.9±3.9 s (21.8%)<br>(CVs per individual (n=8) ranged from 6.4% to 26.5%)
North Pacific right whale	Gunshot bout	Gunshot	I	(50): Table 2	Whale NMML 24: 2.4±0.45 s (18.8%)<br>Whale NMML 85: 3.5±1.2 s (34.3%)[8]<br>Whale NMML 15: 3.6±0.8 s (22.2%)<br>Whale NMML 87: 4.3±1.2 s (27.9%)
	Song	Call[9]	I	(51): Table 3	GS3-PU preliminary phrase inter-call interval: 2.6±0.3 s (11.5%)<br>GS3-PU main phrase inter-call interval: 3.9±0.3 s (7.7%)<br>**GS3-PU terminal phrase inter-call interval: 3.8±0.2 s (5.3%)**<br>GS1-PF terminal phrase inter-call interval: 1.0±0.1 s (10%)
			H	(51): Table 3	GS1-PF main phrase inter-call intervals: 1.1±0.1 s (9.1%) and 2.3±0.5 (21.7%)<br>GS2-TP preliminary phrase inter-call intervals: 1.4±0.1 s (7.1%) and 1.6±0.1 s (6.3%)<br>GS2-TP main phrase inter-call intervals: 1.6±0.1 (6.3%), 3.1±0.1 (3.2%), and 3.5±0.2 (5.7%)<br>GS2-TP terminal phrase inter-call interval: 1.5±0.1 (6.7%), 3.1±0.1 (3.2%), and 3.5±0.1 (2.9%)<br>GS4-DG main phrase inter-call intervals: 0.5±0.1 (20.0%), 9.4±0.3 (3.2%), and 5.0±0.1 (2.0%)

5 A sequence of star wars vocalizations "with shorter and more consistent inter-song intervals" (23)

6 A sequence of star wars vocalizations "with relatively long inter-song intervals" (23)

7 A sequence of star wars vocalizations "with the shortest inter-[vocalization] intervals exhibiting a bimodal distribution" (23)

8 Some gunshots were missed due to intermittent audio signal

9 Gunshots, downsweeps, moans, low-frequency pulsive calls, etc.

		Phrase	I	(51)	GS3-PU inter-phrase interval (between preliminary and main phrase): 14.1±2.2 s (15.6%)
North Atlantic right whale	Scream series	Scream	I	(52)[10]: Table 1	Low noise level condition: 17.9±5.06 s (28.3%)<br>Medium noise level condition: 18.5±4.55 s (24.6%)<br>**High noise level condition: 28.1±4.63 s (16.5%)**
Bowhead whale	Song bout[11]	Song	I	(53)	**Inter-upsweep song interval: 32.0±6.0 s (18.8%)**<br>Inter-downsweep song interval: 38.6±9.5 s (24.6%)<br>Inter-mixed song interval: 9.0±2.0 s (22.2%)
				(54)	Inter-screechy song bout interval: 8.7±2.4 s (27.6%)
Atlantic spotted dolphin	Synchronized scream series	Scream	I	(38): Table 2	Inter-scream interval: 0.40±0.22 s (55.0%)
	Synchronized squawk series	Squawk	I	(38): Table 2	Inter-squawk interval (adults, intraspecific aggression): 0.36±0.33 s (91.7%)<br>Inter-squawk interval (juveniles, intraspecific aggression): 0.64±0.50 s (78.1%)<br>Inter-squawk interval (adults, interspecific aggression): 0.60±0.53 s (88.3%)<br>**Inter-squawk interval (adults, interspecific aggression, during period of maximal physical synchrony): 0.28±0.02 s (7.1%)**[12]
Indo-Pacific bottlenose dolphin	Disconnected multi-loop signature whistles	Loop	I	(55): Figure 4.4A	**Inter-loop interval CVs range from 30.8% (North Zanzibar)** to 47.9% (east coast of Scotland) across eight locations
	Pop train	Pop	I	(25): supplement[13]	Average inter-pop interval CV calculated from 279 trains: 36.3%
	Pop train sequence	Pop train	I	(25): supplement	Average inter-train interval CV calculated from 414 inter-train intervals: 34.8%
Common bottlenose dolphin	Synchronized bray/buzz bout series	Bout[14]	I	(38): Table 2	Inter-bout interval: 0.76±0.20 s (26.3%)
	Bray-call sequence	Element[15]	I	(56): Table 4	Several isochronous sequence types, **including sequence type 7 (0.17±0.0 s, 0%)** and sequence type 11 (0.65±0.2 s, 30.8%)
			H	(56): Table 4	Several heterochronous sequence types, including sequence type 2 (interval 1: 0.11±0.0 s, 0%; interval 2: 0.44±0.16 s, 36.4%) and sequence type 3 (interval 1: 0.14±0.0 s, 0%, interval 2: 0.40±0.10 s, 25%)
	Synchronized buzz bout series	Buzz	I	(38): Table 2	Inter-buzz interval: 3.02±0.88 s (29.1%)

[10] Experiment involved recording screams from surface active groups while playing back various levels of noise

[11] "continuous repetitions of identical song" (53)

[12] Most precise squawk isochrony recorded

[13] Calculated from ESM spreadsheet "full_dataset_for_linear_regression.xlsx", available at https://doi.org/10.5061/dryad.r2280gb9h

[14] A single bray and buzz with no silent interval

[15] Elements include gulps, pops, grunts, cracks, squeaks, and low-frequency narrowband sounds

	Disconnected multi-loop signature whistles	Loop	I	(57): Table 2	Average whistle inter-loop interval CV calculated from 16 animals was 21.1% (ranged from 9% to 46%)
	Synchronized whistle/buzz bout series	Call[16]	H	(38): Table 2	Whistle-buzz interval: 0.22±0.07 s (31.8%)<br>Buzz-whistle interval: 0.84±0.15 s (17.9%)
		Bout[17]	I	(38): Table 2	Inter-bout interval: 0.84±0.15 s (17.9%)
Northern right whale dolphin	Burst pulse unit[18]	Click	I	(58): Table 2	**Inter-click interval: 1.15±0.4 ms (34.8%)**
	Burst pulse series[19]	Burst pulse unit	H	(58): Table 3	Inter-burst pulse unit intervals for burst pulse series #4:<br>A-B interval: 0.115±0.008 s (7.0%)<br>B-C interval: 0.039±0.004 s (10.3%)<br>C-D interval: 0.085±0.009 s (10.6%)<br>D-E interval: 0.073±0.006 s (8.2%)<br>E-F interval: 0.114±0.007 s (6.1%)<br>F-G interval: 0.071±0.003 s (4.2%)<br>G-H interval: 0.088±0.005 s (5.7%)<br>H-I interval: 0.134±0.013 s (9.7%)<br>I-J interval: 0.209±0.011 s (5.3%)
Narwhal	Pulsed call series	Call[20]	I	(59): supplement	Inter-A-call interval (type II vocal sequence): 5.222±1.351 s (25.9%)<br>**Inter-A-call interval (type IX vocal sequence): 2.611±0.367 s (14.1%)**
			H	(59): supplement	A-B interval CVs range from 12.8% to 37.6% and B-A interval CVs range from 8.0% to 96.4% for ten different paired sequences of A and B calls. For example:<br>A-B interval (type VII call sequence): 0.453±0.058 s (12.8%)<br>B-A interval (type VII call sequence): 2.524±0.443 s (17.6%)<br>A-B interval (type X call sequence): 0.335±0.075 s (22.4%)<br>B-A interval (type X call sequence): 5.402±1.204 s (22.3%)
Blainville's beaked whale	Echolocation	Click	I	(60)	**Inter-click interval: 0.37±0.10 s (27.0%)**
Northern bottlenose whale	Echolocation	Click	I	(61)	**Inter-click interval: 0.40±0.05 s (12.5%)**
Cuvier's beaked whale	Echolocation	Click	I	(62)	Inter-click interval: 0.444±0.092 s (20.7%)
				(63)	Inter-click interval (whale A): 0.43±0.092 s (21.4%)<br>**Inter-click interval (whale B): 0.40±0.074 s (18.5%)**

16 A whistle or a buzz

17 A single whistle and buzz separated by a silent interval

18 "composed of between 2 and 159 individual clicks"

19 "composed of 6-18 individual burst-pulse units"

20 Two types of calls (A and B)

Sperm whale	Echolocation	Click	I	(64): Table 2	**Inter-onset interval of clicks: 0.46±0.1 s (21.7%)**
	Surface click series	Surface click[21]	I	(26): Table 1	Inter-surface click interval: 5.47 s (29%)[22]

[21] Referred to by different names in the literature (e.g. clangs (65), slow clicks (66), surface clicks (26))

[22] The standard deviation was not provided, but the CV was.

Supplemental references

1. D. Gerhard, Silence as a cue to rhythm in the analysis of speech and song. *Canadian Acoustics* **31**, 22–23 (2003).

2. D. Margoliash, Acoustic parameters underlying the responses of song-specific neurons in the white-crowned sparrow. *Journal of Neuroscience* **3**, 1039–1057 (1983).

3. H. Williams, K. Staples, Syllable chunking in zebra finch (Taeniopygia guttata) song. *Journal of Comparative Psychology* **106**, 278 (1992).

4. L. S. Burchardt, M. Picciulin, E. Parmentier, M. Bolgan, A primer on rhythm quantification for fish sounds: a Mediterranean case study. *Royal Society Open Science* **8**, 210494 (2021).

5. G. Madison, B. Merker, On the limits of anisochrony in pulse attribution. *Psychological research* **66**, 201–207 (2002).

6. B. H. Repp, A microcosm of musical expression. I. Quantitative analysis of pianists' timing in the initial measures of Chopin's Etude in E major. *The Journal of the Acoustical Society of America* **104**, 1085–1100 (1998).

7. J. E. Wallin, Experimental studies of rhythm and time. *Psychological Review* **18**, 100 (1911).

8. A. Ravignani, D. Bowling, W. T. Fitch, Chorusing, synchrony, and the evolutionary functions of rhythm. *Frontiers in Psychology* **5**, 1–15 (2014).

9. M. Cantor, H. Whitehead, S. Gero, L. Rendell, Cultural turnover among Galápagos sperm whales. *Royal Society Open Science* **3**, 160615 (2016).

10. L. Sayigh, *et al.*, The Watkins Marine Mammal Sound Database: An online, freely accessible resource in *Proceedings of Meetings on Acoustics*, (2016), p. 040013.

11. A. D. Patel, Musical rhythm, linguistic rhythm, and human evolution. *Music Perception* **24**, 99–104 (2006).

12. A. D. Patel, Vocal learning as a preadaptation for the evolution of human beat perception and synchronization. *Philosophical Transactions of the Royal Society B* **376**, 20200326 (2021).

13. V. M. Janik, M. Knörnschild, Vocal production learning in mammals revisited. *Philosophical Transactions of the Royal Society B* **376**, 20200244 (2021).

14. A. M. MacLarnon, G. P. Hewitt, The evolution of human speech: The role of enhanced breathing control. *American Journal of Physical Anthropology: The Official Publication of the American Association of Physical Anthropologists* **109**, 341–363 (1999).

15. M. Larsson, Self-generated sounds of locomotion and ventilation and the evolution of human rhythmic abilities. *Animal cognition* **17**, 1–14 (2014).

16. M. Niarchou, *et al.*, Unravelling the genetic architecture of musical rhythm. *bioRxiv* (2019).

17. A. Fahlman, M. J. Moore, D. Garcia-Parraga, Respiratory function and mechanics in pinnipeds and cetaceans. *Journal of Experimental Biology* **220**, 1761–1773 (2017).

18. W. T. Fitch, Production of vocalizations in mammals. *Visual Communication* **3**, 145 (2006).

19. C. Darwin, *The descent of man, and selection in relation to sex* (D. Appleton, 1872).

20. G. Miller, *Evolution of human music through sexual selection* (na, 2000).

21. J. N. Schneider, E. Mercado III, Characterizing the rhythm and tempo of sound production by singing whales. *Bioacoustics* **28**, 239–256 (2019).

22. E. C. Garland, L. Rendell, L. Lamoni, M. M. Poole, M. J. Noad, Song hybridization events during revolutionary song change provide insights into cultural transmission in humpback whales. *Proceedings of the National Academy of Sciences* **114**, 7822–7829 (2017).

23. J. Gedamke, "Minke whale song, spacing, and acoustic communication on the Great Barrier Reef, Australia," University of California Santa Cruz. (2004).

24. E. M. Oleson, *et al.*, Behavioral context of call production by eastern North Pacific blue whales. *Marine Ecology Progress Series* **330**, 269–284 (2007).

25. B. L. Moore, R. C. Connor, S. J. Allen, M. Krützen, S. L. King, Acoustic coordination by allied male dolphins in a cooperative context. *Proceedings of the Royal Society B* **287**, 20192944 (2020).

26. N. Jaquet, S. Dawson, L. Douglas, Vocal behavior of male sperm whales: Why do they click? *The Journal of the Acoustical Society of America* **109**, 2254–2259 (2001).

27. E. Dissanayake, An ethological view of music and its relevance to music therapy. *Nordic Journal of Music Therapy* **10**, 159–175 (2001).

28. J. G. Roederer, The search for a survival value of music. *Music perception* **1**, 350–356 (1984).

29. H. Whitehead, J. Mann, "Female reproductive strategies of cetaceans" in *Cetacean Societies: Field Studies of Dolphins and Whales*, (Univ of Chicago Press, 2000), pp. 219–246.

30. D. L. Grieser, P. K. Kuhl, Maternal speech to infants in a tonal language: Support for universal prosodic features in motherese. *Developmental psychology* **24**, 14 (1988).

31. R. E. Burnham, D. A. Duffus, Maternal behaviors of gray whales (Eschrichtius robustus) on a summer foraging site. *Marine Mammal Science* **36**, 1212–1230 (2020).

32. L. S. Sayigh, *et al.*, Bottlenose dolphin mothers modify signature whistles in the presence of their own calves. *Proceedings of the National Academy of Sciences* **120**, e2300262120 (2023).

33. B. H. Merker, G. S. Madison, P. Eckerdal, On the role and origin of isochrony in human rhythmic entrainment. *Cortex* **45**, 4–17 (2009).

34. S. Brown, Evolutionary models of music: From sexual selection to group selection. *Perspectives in ethology: Evolution, culture, and behavior*, 231–281 (2000).

35. R. A. Dunlop, "Humpback whales: A seemingly socially simple whale with communicative complexity" in *Ethology and Behavioral Ecology of Mysticetes*, (Springer, 2022), pp. 223–246.

36. H. Whitehead, L. Rendell, *The cultural lives of whales and dolphins* (University of Chicago Press, 2014).

37. M. N. Weiss, S. Ellis, D. P. Croft, Diversity and consequences of social network structure in toothed whales. *Frontiers in Marine Science* **8**, 688842 (2021).

38. D. Herzing, Synchronous and rhythmic vocalizations and correlated underwater behavior of free-ranging Atlantic spotted dolphins (Stenella frontalis) and bottlenose dolphins (Tursiops truncatus) in the Bahamas. *Animal Behavior and Cognition* **2**, 14–29 (2015).

39. J. Zamorano-Abramson, M. Michon, M. Hernández-Lloreda, F. Aboitiz, Multimodal imitative learning and synchrony in cetaceans. A model for human language and music evolution. *Frontiers in Psychology*, 1–17 (2023).

40. M. L. Rekdahl, *et al.*, Non-song social call bouts of migrating humpback whales. *The Journal of the Acoustical Society of America* **137**, 3042–3053 (2015).

41. M. R. McGowen, *et al.*, Phylogenomic resolution of the cetacean tree of life using target sequence capture. *Systematic Biology* **69**, 479–501 (2020).

42. S. Cerchio, *et al.*, Omura's whales (Balaenoptera omurai) off northwest Madagascar: Ecology, behaviour and conservation needs. *Royal Society Open Science* **2**, 150301 (2015).

43. R. Payne, S. McVay, Songs of humpback whales. *Science* **173**, 585–597 (1971).

44. D. K. Mellinger, C. W. Clark, Blue whale (Balaenoptera musculus) sounds from the North Atlantic. *The Journal of the Acoustical Society of America* **114**, 1108–1119 (2003).

45. M. A. McDonald, S. L. Mesnick, J. A. Hildebrand, Biogeographic characterisation of blue whale song worldwide: Using song to identify populations. *Journal of Cetacean Research and Management* **8**, 55–65 (2006).

46. K. M. Stafford, S. L. Nieukirk, C. G. Fox, Geographic and seasonal variation of blue whale calls in the North Pacific. *Journal of Cetacean Research and Management* **3**, 65–76 (2001).

47. E. Mercado III, S. Handel, Understanding the structure of humpback whale songs (L). *The Journal of the Acoustical Society of America* **132**, 2947–2950 (2012).

48. S. Handel, S. K. Todd, A. M. Zoidis, Rhythmic structure in humpback whale (Megaptera novaeangliae) songs: Preliminary implications for song production and perception. *The Journal of the Acoustical Society of America* **125**, EL225–EL230 (2009).

49. J. Gedamke, D. P. Costa, A. Dunstan, Localization and visual verification of a complex minke whale vocalization. *The Journal of the Acoustical Society of America* **109**, 3038–3047 (2001).

50. J. L. Crance, C. L. Berchok, J. L. Keating, Gunshot call production by the North Pacific right whale Eubalaena japonica in the southeastern Bering Sea. *Endangered Species Research* **34**, 251–267 (2017).

51. J. L. Crance, C. L. Berchok, D. L. Wright, A. M. Brewer, D. F. Woodrich, Song production by the North Pacific right whale, Eubalaena japonica. *The Journal of the Acoustical Society of America* **145**, 3467–3479 (2019).

52. S. E. Parks, C. W. Clark, P. L. Tyack, Short- and long-term changes in right whale calling behavior: The potential effects of noise on acoustic communication. *The Journal of the Acoustical Society of America* **122**, 3725–3731 (2007).

53. J. Delarue, M. Laurinolli, B. Martin, Bowhead whale (Balaena mysticetus) songs in the Chukchi Sea between October 2007 and May 2008. *The Journal of the Acoustical Society of America* **126**, 3319–3328 (2009).

54. K. M. Stafford, S. E. Moore, K. L. Laidre, M. P. Heide-Jørgensen, Bowhead whale springtime song off West Greenland. *The Journal of the Acoustical Society of America* **124**, 3315–3323 (2008).

55. T. Gridley, "Geographic and species variation in bottlenose dolphin (Tursiops spp.) signature whistle types," University of St. Andrews. (2010).

56. D. S. Pace, *et al.*, Bray-call sequences in the Mediterranean common bottlenose dolphin (Tursiops truncatus) acoustic repertoire. *Biology* **11**, 367 (2022).

57. H. C. Esch, L. S. Sayigh, R. S. Wells, Quantifying parameters of bottlenose dolphin signature whistles. *Marine Mammal Science* **25**, 976–986 (2009).

58. S. Rankin, J. Oswald, J. Barlow, M. Lammers, Patterned burst-pulse vocalizations of the northern right whale dolphin, Lissodelphis borealis. *The Journal of the Acoustical Society of America* **121**, 1213–1218 (2007).

59. S. Walmsley, "Rhythmically repeated patterns of pulsed vocalizations in wild narwhals (Monodon monoceros)," University of St. Andrews. (2018).

60. M. Johnson, P. T. Madsen, W. M. X. Zimmer, N. Aguilar De Soto, P. L. Tyack, Foraging Blainville's beaked whales (Mesoplodon densirostris) produce distinct click types matched to different phases of echolocation. *Journal of Experimental Biology* **209**, 5038–5050 (2006).

61. S. K. Hooker, H. Whitehead, Click characteristics of northern bottlenose whales (Hyperoodon ampullatus). *Marine Mammal Science* **18**, 69–80 (2002).

62. A. Frantzis, J. C. Goold, E. K. Skarsoulis, M. I. Taroudakis, V. Kandia, Clicks from Cuvier's beaked whales, Ziphius cavirostris (L). *The Journal of the Acoustical Society of America* **112**, 34–37 (2002).

63. W. M. X. Zimmer, M. P. Johnson, P. T. Madsen, P. L. Tyack, Echolocation clicks of free-ranging Cuvier's beaked whales (Ziphius cavirostris). *The Journal of the Acoustical Society of America* **117**, 3919–3927 (2005).

64. L. S. Burchardt, M. Knörnschild, Comparison of methods for rhythm analysis of complex animals' acoustic signals. *PLoS Computational Biology* **16** (2020).

65. J. C. D. Gordon, Sperm whale groups and social behaviour observed off Sri Lanka. *Report of the International Whaling Commission* **37**, 205–217 (1987).

66. L. S. Weilgart, H. Whitehead, Distinctive vocalizations from mature male sperm whales (Physeter macrocephalus). *Canadian Journal of Zoology* **66**, 1931–1937 (1988).

67. E. M. Oleson, J. Barlow, J. Gordon, S. Rankin, J. A. Hildebrand, Low frequency calls of Bryde's whales. *Marine Mammal Science* **19**, 407–419 (2003).

68. S. Cerchio, C. R. Weir, Mid-frequency song and low-frequency calls of sei whales in the Falkland Islands (2022).

69. J. Delarue, S. K. Todd, S. M. Van Parijs, L. Di Iorio, Geographic variation in Northwest Atlantic fin whale (Balaenoptera physalus) song: Implications for stock structure assessment. *The Journal of the Acoustical Society of America* **125**, 1774–1782 (2009).

70. A. Pereira, D. Harris, P. Tyack, L. Matias, Fin whale acoustic presence and song characteristics in seas to the southwest of Portugal. *The Journal of the Acoustical Society of America* **147**, 2235–2249 (2020).

71. A. Širović, E. M. Oleson, J. Buccowich, A. Rice, A. R. Bayless, Fin whale song variability in southern California and the Gulf of California. *Scientific Reports* **7**, 1–11 (2017).

72. S. Cerchio, M. Dahlheim, Variation in feeding vocalizations of humpback whales Megaptera novaeangliae from southeast Alaska. *Bioacoustics* **11**, 277–295 (2001).

73. L. N. Guinee, K. B. Payne, Rhyme-like repetitions in songs of humpback whales. *Ethology* **79**, 295–306 (1988).

74. P. O. Thompson, W. A. Friedl, A long term study of low frequency sounds from several species of whales off Oahu, Hawaii. *Cetology* **45**, 1–19 (1982).

75. J. L. Crance, C. L. Berchok, J. L. Keating, Gunshot call production by the North Pacific right whale Eubalaena japonica in the southeastern Bering Sea. *Endangered Species Research* **34**, 251–267 (2017).

76. M. E. dos Santos, A. J. F. Ferreira, S. Harzen, “Rhythmic sound sequences emitted by aroused bottlenose dolphins in the Sado estuary, Portugal” in *Sensory Systems of Aquatic Mammals*, R. A. Kastelein, J. A. Thomas, P. E. Nachtigall, Eds. (De Spil Publishers, 1995), pp. 325–334.

77. M. F. N. R. Vargas, “Revealing structure in vocalisations of parrots and social whales,” Georg-August-Universität Göttingen. (2017).

78. E. M. J. Zwamborn, H. Whitehead, Repeated call sequences and behavioural context in long-finned pilot whales off Cape Breton, Nova Scotia, Canada. *Bioacoustics* **26**, 169–183 (2017).

79. P. J. O. Miller, A. D. Shapiro, P. L. Tyack, A. R. Solow, Call-type matching in vocal exchanges of free-ranging resident killer whales, Orcinus orca. *Animal Behaviour* **67**, 1099–1107 (2004).

80. A. E. Simonis, *et al.*, High-frequency modulated signals of killer whales (Orcinus orca) in the North Pacific. *The Journal of the Acoustical Society of America* **131**, EL295–EL301 (2012).

81. O. Le Bot, J. I. Mars, C. Gervaise, Y. Simard, Rhythmic analysis for click train detection and source separation with examples on beluga whales. *Applied Acoustics* **95**, 37–49 (2015).

82. T. M. Schulz, H. Whitehead, S. Gero, L. Rendell, Overlapping and matching of codas in vocal interactions between sperm whales: Insights into communication function. *Animal Behaviour* **76**, 1977–1988 (2008).

83. L. Rendell, H. Whitehead, Vocal clans in sperm whales (Physeter macrocephalus). *Proceedings of the Royal Society of London Series B: Biological Sciences* **270**, 225–31 (2003).
